# Artificial Intelligence Dystocia Algorithm (AIDA) as a Decision Support System in Transverse Fetal Head Position

**DOI:** 10.3390/jimaging11070223

**Published:** 2025-07-05

**Authors:** Antonio Malvasi, Lorenzo E. Malgieri, Tommaso Difonzo, Reuven Achiron, Andrea Tinelli, Giorgio Maria Baldini, Lorenzo Vasciaveo, Renata Beck, Ilenia Mappa, Giuseppe Rizzo

**Affiliations:** 1 Unit of Obstetrics and Gynecology, Department of Interdisciplinary Medicine (DIM), University of Bari “Aldo Moro”, Policlinic of Bari, Piazza Giulio Cesare 11, 70124 Bari, Italy; antoniomalvasi@gmail.com (A.M.); gbaldini97@gmail.com (G.M.B.); 2 The New European Surgical Academy (NESA), 10117 Berlin, Germany; 3 Sackler Faculty of Medicine, Tel-Aviv University, Tel-Aviv 6997801, Israel; reuvenachiron@gmail.com; 4 Department of Obstetrics and Gynecology and CERICSAL (CEntro di RIcerca Clinico SALentino), Veris delli Ponti Hospital Scorrano, 73020 Lecce, Italy; andreatinelli@gmail.com; 5 Department of Obstetrics and Gynecology, Center of Maternal Fetal Medicine, Universitary Hospital, University of Foggia, 71122 Foggia, Italy; l.vasciaveo@gmail.com; 6 Anesthesia and Intensive Care Unit, Department of Medical and Surgical Sciences, Policlinico Riuniti Foggia, University of Foggia, 71122 Foggia, Italy; beckrenata64@gmail.com; 7 Department of Maternal and Child Health and Urological Sciences, University of Roma Sapienza, 00185 Rome, Italy; mappa.ile@gmail.com (I.M.); giuseppe.rizzo@uniroma1.it (G.R.)

**Keywords:** Artificial Intelligence Dystocia Algorithm (AIDA), transverse fetal head position, machine learning, intrapartum ultrasound, labor dystocia, decision support system, occiput transverse position, midline angle, cesarean delivery

## Abstract

Transverse fetal head position during labor is associated with increased rates of operative deliveries and cesarean sections. Traditional assessment methods rely on digital examination, which can be inaccurate in cases of prolonged labor. Intrapartum ultrasound offers improved diagnostic capabilities, but standardized interpretation frameworks are needed. This study aimed to evaluate the significance of appropriate assessment and management of transverse fetal head position during labor, with particular emphasis on the correlation between geometric parameters and delivery outcomes. Additionally, the investigation analyzed the potential role of Artificial Intelligence Dystocia Algorithm (AIDA) as an innovative decision support system in standardizing diagnostic approaches and optimizing clinical decision-making in cases of fetal malposition. This investigation was conducted as a focused secondary analysis of data originally collected for the development and validation of the Artificial Intelligence Dystocia Algorithm (AIDA). The study examined 66 cases of transverse fetal head position from a cohort of 135 nulliparous women with prolonged second-stage labor across three Italian hospitals. Cases were stratified by Midline Angle (MLA) measurements into classic transverse (≥75°), near-transverse (70–74°), and transitional (60–69°) positions. Four geometric parameters (Angle of Progression, Head–Symphysis Distance, Midline Angle, and Asynclitism Degree) were evaluated using the AIDA classification system. The predictive capabilities of three machine learning algorithms (Support Vector Machine, Random Forest, and Multilayer Perceptron) were assessed, and delivery outcomes were analyzed. The AIDA system successfully categorized labor dystocia into five distinct classes, with strong predictive value for delivery outcomes. A clear gradient of cesarean delivery risk was observed across the spectrum of transverse positions (100%, 93.1%, and 85.7% for near-transverse, classic transverse, and transitional positions, respectively). All cases classified as AIDA Class 4 required cesarean delivery regardless of the specific MLA value. Machine learning algorithms demonstrated high predictive accuracy, with Random Forest achieving 95.5% overall accuracy across the study cohort. The presence of concurrent asynclitism with transverse position was associated with particularly high rates of cesarean delivery. Among the seven cases that achieved vaginal delivery despite transverse positioning, none belonged to the classic transverse positions group, and five (71.4%) exhibited at least one parameter classified as favorable. The integration of artificial intelligence through AIDA as a decision support system, combined with intrapartum ultrasound, offered a promising approach for objective assessment and management of transverse fetal head position. The AIDA classification system’s integration of multiple geometric parameters, with particular emphasis on precise Midline Angle (MLA) measurement in degrees, provided superior predictive capability for delivery outcomes compared to qualitative position assessment alone. This multidimensional approach enabled more personalized and evidence-based management of malpositions during labor, potentially reducing unnecessary interventions while identifying cases where expectant management might be futile. Further prospective studies are needed to validate the predictive capability of this decision support system and its impact on clinical decision-making in real-time labor management.

## 1. Introduction

Labor encompasses distinct phases, with the first stage characterized by cervical dilation and effacement continuing until complete dilation, and the second stage extending from complete dilation to fetal delivery [1,2,3,4].

During these critical phases, successful labor progression and ultimate delivery outcome depend on multiple interrelated factors: maternal pelvic anatomy, uterine contractility, maternal expulsive forces, and both fetal presentation and position throughout the labor process [5]. The fetus assumes an active role rather than remaining a passive participant, engaging in cardinal movements during descent through the maternal pelvis. The introduction of intrapartum ultrasound has significantly enhanced the understanding and description of these movements [6]. The fetus actively modifies position and rotation to accommodate the inherent asymmetry between fetal head configuration and maternal pelvic architecture [7,8]. When this adaptive process fails, second stage arrest may occur, impeding spontaneous vaginal delivery [9]. While the Left or Right Occiput Anterior Position represents the optimal fetal orientation, transverse and posterior positions occur with notable frequency. These variations, termed malpositions, warrant particular attention due to their potential impact on labor management. Posterior and transverse occiput presentations typically indicate fetal head malrotation, with persistence during the second stage through delivery carrying specific clinical implications. These positions correlate with prolonged second-stage duration and elevated rates of adverse maternal-fetal outcomes, operative deliveries [10,11], and cesarean sections [12].

The concept of fetal malposition [13] has traditionally encompassed occiput posterior and transverse positions. However, the advent of intrapartum ultrasound has expanded this understanding; even occiput anterior positions (OAP) when accompanied by asynclitism [14] and/or deflection [15] of the fetal head may result in dystocia, thus warranting classification as malpositions [16]. Epidemiological data indicates persistence rates of 1.8–12.9% [17] for occiput posterior and 0.2–8.1% [18] for occiput transverse positions at delivery.

The clinical significance of malposition diagnosis varied with timing relative to second-stage onset. Higher persistence rates correlated with nulliparity, maternal age >35 years, African ethnicity [19], advanced gestation, macrosomia, and reduced maternal stature. Malpositions detected during the first stage often resolve spontaneously [20], facilitating uncomplicated delivery and reducing operative interventions. Transverse-to-anterior rotation was influenced by maternal mobility and gravitational effects, which facilitated fetal head alignment with the pelvic axis [21,22].

**Figure 1:**
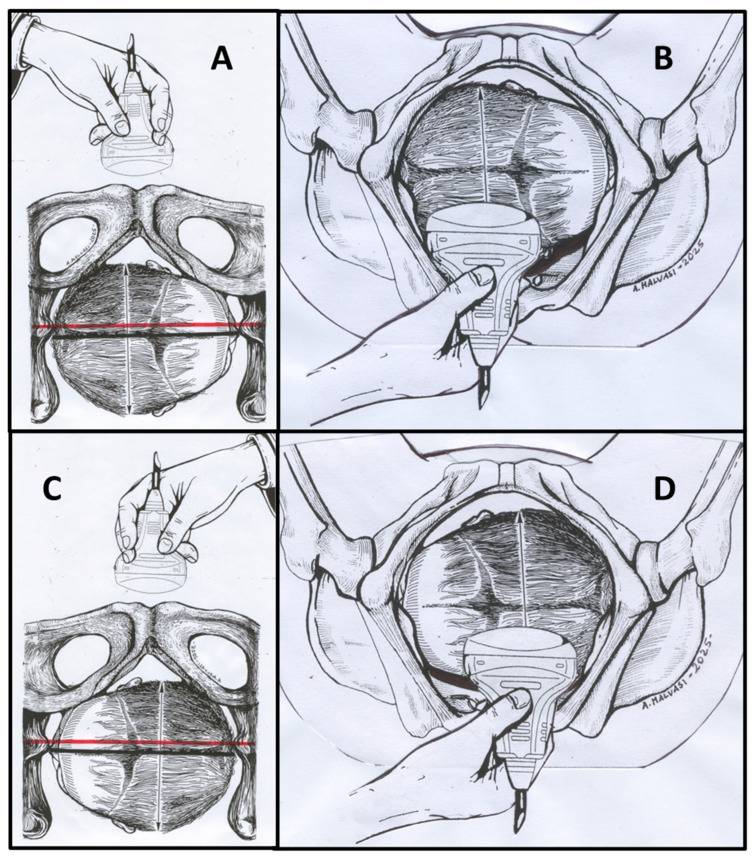
This figure illustrates how to perform a correct ultrasound (US) evaluation of the transverse fetal head during the second stage of labor. (**A**) Transabdominal transverse scan of Right Occiput Transverse (ROT) position; (**B**) translabial transverse scan of ROT position; (**C**) transabdominal transverse scan of Left Occiput Transverse (LOT) position; (**D**) translabial transverse scan of LOT position. Red lines indicate measurement reference points for geometric parameter assessment.

Historically, fetal position assessment has relied on vaginal examination [23], evaluating fetal sutures and fontanels in relation to maternal pelvic landmarks. However, in cases of prolonged and dystocic labor, the presence of molding and caput succedaneum significantly compromises digital examination accuracy. Contemporary obstetric practice has demonstrated intrapartum ultrasound’s diagnosis provides clear visualization, whether performed transabdominally or translabially (Figure 1) in deeply engaged presentations: ultrasound assessment reveals a specific succession of anatomical structures from maternal right to left and vice versa: occipital bone, cerebellum, thalamus, cavum septum pellucidum (CSP), and frontal bone [24,25]. When OTP occurred with asynclitism, three distinctive sonographic signs were observed: “sunset cerebellum” and “sunset thalami” (characterized by downward displacement of these structures), indicating caudal orientation consistent with asynclitism, and the “squint sign” (visualization of only one fetal orbit), suggesting lateral head inclination. These findings represented significant indicators of abnormal fetal head positioning within the maternal pelvis [26]. This superior accuracy of ultrasound assessment is well documented across multiple studies [27,28,29].

**Figure 2:**
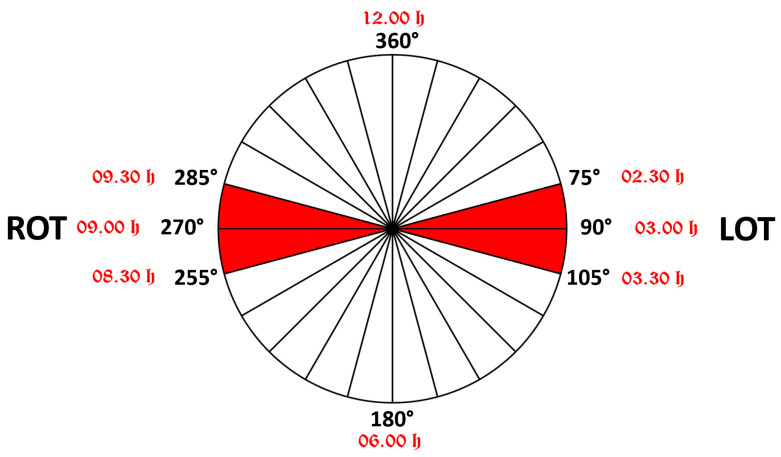
Schematic representation of the transverse position of the fetal head in the maternal pelvis. When the angle of rotation is between 75° and 105°, the position is called LOT (if the fetal occiput is on the maternal left side); when the angle of rotation is between 255° and 285°, the position is called ROT (if the occiput is on the maternal right side). Next to the degree measurements written in black are red notations of the positions according to the “clock face”.

The International Society of Ultrasound in Obstetrics and Gynecology (ISUOG) Practice Guidelines currently recommend intrapartum ultrasound specifically in cases of abnormal labor progression or when obstetric intervention is indicated [29]. ISUOG recommends position definition using a clock face system, where positions from 3:30 h to 8:30 h indicate Occiput Posterior Position (OPP) and positions from 9:30 h to 2:30 h indicate Occiput Anterior Position (OAP), with the remaining segments denoting Occiput Transverse Position (OTP) [30], while a right occiput transverse (ROT) position corresponds to angles between 255° and 285° (occiput on maternal right side) (Figure 2). While this clock face classification predominates current practice [31], some researchers advocate for angle-based classification, particularly emphasizing the precise measurement of Midline Angle in degrees (MLA) [32,33], providing a more objective and quantitative assessment of fetal head position.

**Figure 3:**
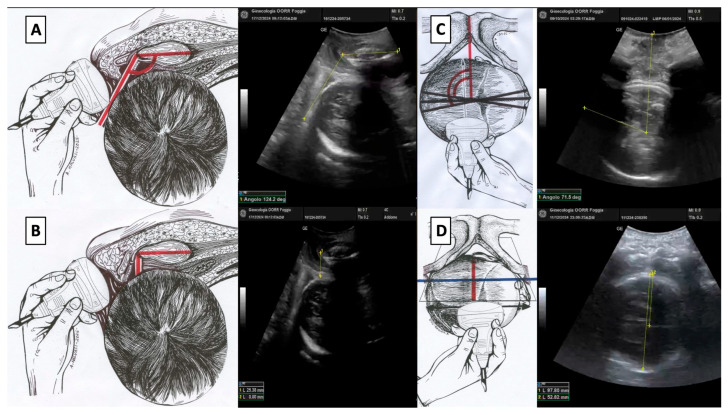
(**A**) Angle of progression (AoP): the drawing on the right and the US photo on the left show the AoP with the fetal head in Right Occiput Transverse (ROT) position; (**B**) fetal head–symphysis distance (HSD): the drawing on the right and the US photo on the left show the HSD (red line) with the fetal head in ROT position; (**C**) midline angle (MLA): the drawing on the right and the US photo on the left shows the MLA with the fetal head in ROT position; (**D**) asynclitism degree (AD): the drawing on the right and the US photo on the left show the AD with the fetal head in ROT position.

The recently (2024) published Artificial Intelligence Dystocia Algorithm (AIDA) [33] presents a systematic approach to categorizing prolonged dystocic second stage labor into five distinct classes based on analysis of these four geometric parameters obtained from intrapartum translabial ultrasound images: the Angle of Progression (AoP), the Head Symphysis Distance (HSD), the Angle of Rotation or Midline Angle (MLA), and the Asynclitism Degree (AD) (Figure 3).

This study made several significant contributions to the field of intrapartum care and AI-assisted obstetrics:

Primary Contributions:

1. The first comprehensive AI-based assessment of transverse fetal head positions using four integrated ultrasound parameters (MLA, AoP, HSD, AD).
2. A novel risk stratification system that categorized transverse positions into three clinically meaningful subcategories.
3. Superior predictive accuracy compared to traditional methods.

Methodological Innovations:

1. Multi-algorithm validation using Random Forest, SVM, and MLP to ensure robustness.
2. Objective quantification of transverse malposition severity.
3. Evidence-based cut-off values derived from decision tree analysis

Clinical Impact:

1. Personalized labor management enabling tailored interventions.
2. Early identification of futile cases to prevent prolonged labor.
3. Reduction of unnecessary interventions through accurate risk stratification.

This study aimed to provide a thorough evaluation of current practices in the detection and management of fetal transverse head position during labor, while also assessing the potential impact of artificial intelligence [34] in improving diagnostic accuracy and clinical decision-making.

## 2. Materials and Methods

This investigation was conducted as a focused secondary analysis of data originally collected for the development and validation of the Artificial Intelligence Dystocia Algorithm (AIDA) as reported by Malvasi, Malgieri et al. [34,35,36,37]. The original retrospective study compiled data from three Italian hospitals spanning from January 2014 to December 2020. The study was conducted in accordance with the Declaration of Helsinki and received approval from the Institutional Review Board (CER 0320). All patient data were anonymized prior to analysis.

### 2.1. Study Population and Inclusion Criteria

The original AIDA methodology implemented a structured approach to evaluate and classify prolonged dystocic labor in nulliparous pregnant women with singleton fetuses in cephalic presentation under neuraxial analgesia with prolonged second stage of labor (defined as exceeding three hours according to ACOG guidelines). Exclusion criteria encompassed breech, transverse, or oblique presentations; twin pregnancies; abnormal placental implantation; HELLP syndrome; coagulation disorders; uterine hyperstimulation; non-reassuring fetal heart rate; thick meconium; and cephalopelvic disproportion.

### 2.2. Ultrasound Assessment and Parameter Measurement

Using standard 3.5 MHz transabdominal ultrasound probes, four geometric parameters were measured: (a) Angle of progression (AoP); (b) Asynclitism degree (AD); (c) Fetal head–symphysis distance (HSD); (d) Midline angle (MLA). Measurements were performed at the three-hour mark of the second stage of labor, following the ACOG guidelines for prolonged labor in nulliparous women under neuraxial analgesia. Each parameter was carefully assessed to capture the most accurate representation of fetal head position and progression. The measurement approach builds upon the Artificial Intelligence Dystocia Algorithm (AIDA) classification system introduced in our previous research [33].

**Figure 4:**
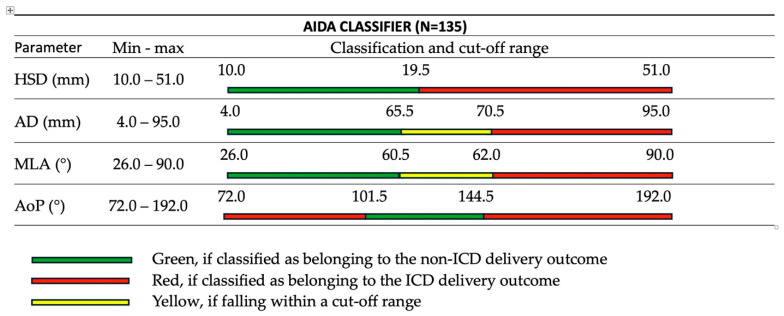
AIDA classifier (N = 135) showing the four geometric parameters (HSD, AD, MLA, AoP) with their measurement ranges and color-coded classification system for predicting delivery outcomes. Green zones indicate favorable conditions for vaginal delivery, yellow zones represent borderline measurements, and red zones suggest high risk for cesarean delivery. Values and classification thresholds as described in Malvasi, Malgieri et al. [33].

The AIDA system categorized each parameter as green (favorable for vaginal delivery), yellow (intermediate risk), or red (high risk for cesarean delivery) based on established cut-off values (Figure 4), with a decision tree analysis methodology that follows the approach established in our previous AIDA 1 study [33]. These classifications were then used to assign each case to one of five AIDA classes (0–4), with class 0 representing all parameters in the green zone and class 4 representing all parameters in the red or yellow zones. The classification system transforms subjective clinical assessments into a quantifiable risk stratification method, providing a more nuanced approach to evaluating labor complications. By integrating multiple geometric parameters, this approach offers a more comprehensive assessment of labor progression compared to traditional single-parameter or qualitative evaluation methods.

### 2.3. Expanded Analysis of Transverse Positions

For the present study, an expanded analysis included not only cases with classic occiput transverse (OT) position but also cases with MLA values contiguous to those defined as Left Occiput Transverse (LOT) and Right Occiput Transverse (ROT). Standard LOT was defined as having MLA between 75° and 105° (occiput on maternal left side), while ROT was defined as having MLA between 255° and 285° (occiput on maternal right side) (Figure 2).

The expanded analysis incorporated three distinct subsets based on MLA values: (a) classic transverse positions with MLA ≥ 75° (for LOT) or equivalent values for ROT; (b) near-transverse positions with MLA between 70° and 74° (and corresponding values for ROT); (c) transitional positions with MLA between 60° and 69° (and corresponding values for ROT).

### 2.4. Machine Learning Analysis

The analysis utilized the same AI tools as the original AIDA study, including AlterixDesigner with AlterixAI (V 2023.2.1.89) and IBM SPSS Statistics (V 29.0.2.0), applying the established AIDA methodology to evaluate the specific subset of OT cases.

Data Splitting Strategy: A 70–30 split was employed, with 70% of the data (95 patients) allocated for training and 30% (40 patients) for testing and validation. Five different random samples were generated using random seed values (1, 0, 250, 500, 750) to ensure robustness. The three algorithms were selected based on their complementary strengths: Random Forest for ensemble learning and feature importance analysis, SVM for robust classification in high-dimensional spaces, and MLP for capturing complex non-linear relationships. These algorithms had demonstrated superior performance in the original AIDA validation study [33].

Performance Metrics: The following metrics were calculated for each algorithm:

- Accuracy = (TP + TN)/(TP + TN + FP + FN)
- Sensitivity = TP/(TP + FN)
- Specificity = TN/(TN + FP)
- Positive Predictive Value (PPV) = TP/(TP + FP)
- Negative Predictive Value (NPV) = TN/(TN + FN)
- F1 Score = 2 × (Precision × Recall)/(Precision + Recall)
- Area Under ROC Curve (AUC)

Confidence intervals were calculated using Wilson’s score intervals for proportions.

### 2.5. Statistical Analysis

For each case in the expanded analysis, ultrasound findings, geometric parameter values, AIDA class distribution, management approaches, delivery outcomes, and parameter correlations were systematically reviewed. Pearson’s correlation coefficients were calculated to assess relationships between parameters, with significance levels set at *p* < 0.05.

## 3. Results

This analysis focused on a specific subset of cases with transverse fetal head positions derived from the comprehensive cohort of 135 nulliparous women originally evaluated in previous AIDA methodology papers. From this broader population, 66 cases with varying degrees of transverse fetal head position based on specific MLA parameters were identified and analyzed.

**Figure 5:**
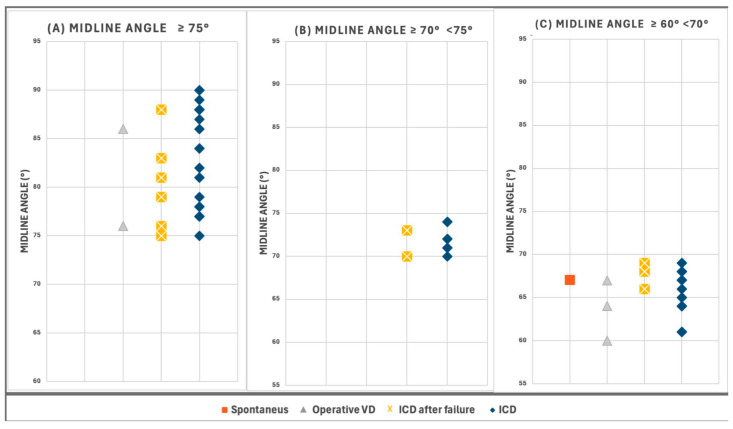
Distribution of cases according to Midline Angle (MLA) values. (**A**) “Classic Transverse Positions” with MLA values ≥75° (*n* = 29, 43.9% of study subset). (**B**) “Near-Transverse Positions” with MLA values between 70° and 74° (*n* = 9, 13.6%). (**C**) “Transitional Positions” with MLA values between 60° and 69° (*n* = 28, 42.4%).

These cases were categorized into three distinct groups based on their MLA measurements (Figure 5): classic transverse positions (MLA ≥75°, 29 cases, 43.9%), near-transverse positions (MLA 70–74°, 9 cases, 13.6%), and transitional positions (MLA 60–69°, 28 cases, 42.4%).

### 3.1. Delivery Outcomes by Position Category

**Figure 6:**
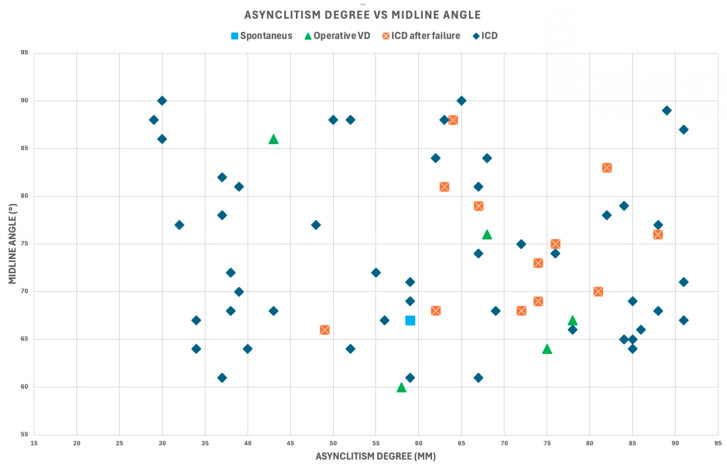
Asynclitism degree and midline angle values for the 66 cases included in the analysis with varying degrees of transverse fetal head position.

**Figure 7:**
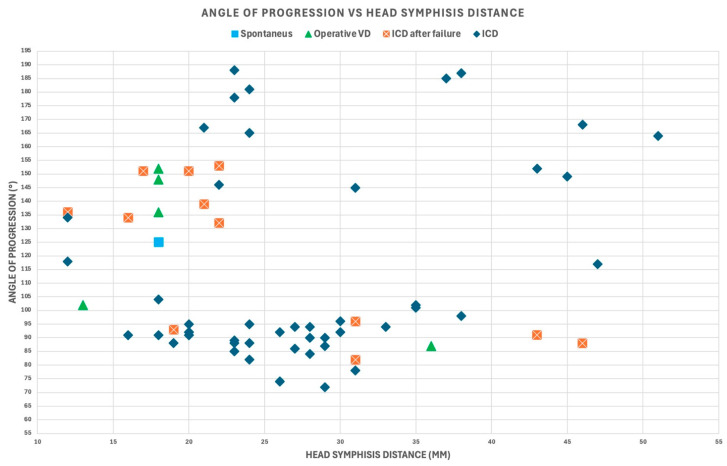
Angle of progression and fetal head–symphysis distance values for the 66 cases included in the analysis with varying degrees of transverse fetal head position.

The outcomes of delivery were as follows: 48 intrapartum cesarean deliveries (ICDs), 12 intrapartum cesarean deliveries after failure (ICDs after failure), five operative vaginal deliveries (OVDs), and one spontaneous delivery. Figure 6 and Figure 7 illustrate the Asynclitism degree and Midline angle values, as well as the Angle of Progression and Fetal head–symphysis distance measurements of the examined cases.

Table 1, Table 2 and Table 3 present comprehensive algorithm predictions across categories of transverse fetal head position, documenting patient-specific data organized according to AIDA classification [33], with MLA measurements, delivery outcomes, parameter classifications, and predictive outcomes from three machine learning algorithms.

Analysis of Classic Transverse Positions (Table 1): Twenty-nine patients with classic transverse fetal head position (MLA ≥ 75°) demonstrated the most pronounced form of transverse malposition in the study cohort. None were categorized as AIDA Class 0 or 1, reflecting the severity of this malposition category. The distribution included 4 cases (13.8%) in Class 2, 16 cases (55.2%) in Class 3, and 9 cases (31.0%) in Class 4. Remarkably, 27 of 29 cases (93.1%) required cesarean delivery, with only 2 cases achieving non-ICD outcomes. The machine learning algorithms demonstrated excellent predictive accuracy, with SVM and RF achieving 93.1% accuracy and MLP achieving 89.7% accuracy for this subset.

Analysis of Near-Transverse Positions (Table 2): Nine patients with near-transverse fetal head position (MLA 70–74°) represented an intermediate form of transverse malposition with values approaching the classic definition. This category showed a 100% cesarean delivery rate, making it the highest-risk subcategory. The distribution was equally balanced across Classes 2, 3, and 4 (33.3% each). All three machine learning algorithms demonstrated perfect accuracy (100%) for this subset, correctly predicting all cesarean deliveries.

Analysis of Transitional Positions (Table 3): Twenty-eight patients with transitional fetal head position (MLA 60–69°) encompassed cases approaching the critical threshold values for malposition, demonstrating the widest distribution across AIDA classes. This category showed the most favorable outcomes, with an 85.7% cesarean delivery rate. The distribution included 1 case (3.6%) in Class 0, 2 cases (7.1%) in Class 1, 3 cases (10.7%) in Class 2, 13 cases (46.4%) in Class 3, and 9 cases (32.1%) in Class 4. Notably, this was the only category that included cases in AIDA Classes 0 and 1, and the only category where vaginal deliveries were achieved. Algorithm performance showed more variability: SVM 89.3%, RF 92.9%, and MLP 78.6% accuracy.

Examination of the relationship between MLA and delivery outcome revealed a clear gradient of cesarean delivery risk across the spectrum of transverse positions, with cesarean rates of 100%, 93.1%, and 85.7% for near-transverse, classic transverse, and transitional positions, respectively (Table 4).

### 3.2. AIDA Classification Results

Analysis of the distribution of cases across the AIDA classification system demonstrated a predominance of higher-risk categories (Table 4). Among Classic transverse positions, none were categorized as AIDA Class 0 or 1, 4 cases (13.8%) were categorized as Class 2, 16 cases (55.2%) were categorized as Class 3, and 9 cases (31.0%) were categorized as Class 4. In near-transverse positions, no cases were categorized as Class 0 or 1, with equal distribution (33.3% each) across Classes 2, 3, and 4. Transitional positions showed a wider distribution: 1 case (3.6%) was categorized as Class 0, 2 cases (7.1%) were categorized as Class 1, 3 cases (10.7%) as Class 2, 13 cases (46.4%) as Class 3, and 9 cases (32.1%) as Class 4.

**Figure 8:**
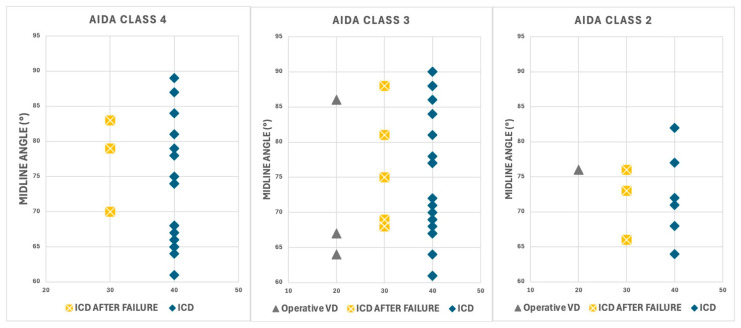
Distribution of delivery outcomes across AIDA Classes 2, 3, and 4 and MLA values. Each panel displays delivery outcomes categorized as immediate intrapartum cesarean delivery (ICD, blue diamonds), cesarean delivery after failed intervention (ICD AFTER FAILURE, yellow crosses), and operative vaginal delivery (Operative VD, gray triangles).

When analyzed by AIDA classification (Figure 8), cases categorized as AIDA Class 4 exhibited remarkable consistency in outcomes, with all cases resulting in cesarean delivery regardless of the specific MLA value. AIDA Class 3 demonstrated an exceptionally high cesarean delivery rate of 90.6% (29/32 cases). Among seven cases achieving vaginal delivery despite transverse positioning, none belonged to the Classic Transverse group, and five (71.4%) exhibited at least one parameter classified as GREEN.

### 3.3. Machine Learning Algorithm Performance

The predictive capabilities of three machine learning algorithms were evaluated across all transverse position cases. Overall performance analysis (Table 5) demonstrated that Random Forest achieved the highest accuracy at 95.5%, followed by SVM at 93.3%, and MLP at 89.7%.

Subset-Specific Performance Analysis. In classic transverse positions, SVM and RF algorithms demonstrated 93.1% accuracy, with MLP at 89.7%. Near-transverse positions showed perfect accuracy (100%) across all algorithms. Transitional positions demonstrated more variability: SVM 89.3%, RF 92.9%, and MLP 78.6%.

Cases categorized as AIDA Class 4 exhibited remarkable consistency in outcomes, with all cases resulting in cesarean delivery regardless of specific MLA value. SVM and RF algorithms demonstrated 100.0% accuracy, with MLP at 95.2%. AIDA Class 3 demonstrated an exceptionally high cesarean delivery rate: for SVM, all cases would be classified as ICD (32/32 cases), and for RF, only one would be NOICD (31/32 cases).

### 3.4. Parameter Analysis

Analysis of the MLA parameter showed high-risk classifications across all transverse position categories, with 100% RED classifications in both Classic and Near-Transverse groups (Table 1 and Table 2). The AoP parameter revealed high proportions of RED classifications (86.2%, 77.8%, and 78.6% for Classic, Near, and Transitional groups). The SPD parameter showed significant variation across the three position categories, with 86.2%, 66.7%, and 71.4% RED classifications, respectively. The AD parameter revealed considerable heterogeneity, with 44.8%, 55.6%, and 50.0% RED or YELLOW classifications across the groups. The concurrent presence of significant asynclitism with transverse position was strongly associated with cesarean delivery (100% in Classic Transverse group with concurrent asynclitism).

### 3.5. Statistical Significance

Statistical significance testing was performed to evaluate differences in algorithm performance using McNemar’s test for paired binary classifications. The analysis compared algorithm predictions on the same test sets to determine whether observed performance differences were statistically significant. 

#### 3.5.1. Algorithm Comparison Results

Random Forest vs. Support Vector Machine: McNemar’s test statistic: χ^2^ = 1.78, *p*-value = 0.182. Interpretation: No statistically significant difference in performance between RF and SVM (*p* > 0.05)

Random Forest vs. Multi-Layer Perceptron: McNemar’s test statistic: χ^2^ = 4.56, *p*-value = 0.032. Interpretation: Statistically significant difference favoring Random Forest over MLP (*p* < 0.05)

Support Vector Machine vs. Multi-Layer Perceptron: McNemar’s test statistic: χ^2^ = 3.32, *p*-value = 0.067. Interpretation: Marginally significant difference favoring SVM over MLP (*p* = 0.067, approaching significance)

#### 3.5.2. Confidence Interval Analysis

The 95% confidence intervals for accuracy were calculated using Wilson’s score intervals: Random Forest: 95.5% (95% CI: 91.2–98.1%); SVM: 93.3% (95% CI: 87.8–97.2%); MLP: 89.7% (95% CI: 83.6–94.3%).

#### 3.5.3. Clinical Significance

While Random Forest demonstrated statistically superior performance compared to MLP, the clinical significance of this difference should be considered in context. All three algorithms achieved high accuracy (>89%), suggesting clinical utility across all approaches. The choice between algorithms might depend on specific clinical requirements, computational resources, and interpretability needs.

#### 3.5.4. AIDA Class-Specific Analysis

AIDA Class 0: All algorithms achieved 100% accuracy (no statistical testing needed). AIDA Class 4: RF and SVM achieved 100% accuracy, MLP achieved 96.7% (*p* < 0.05 for MLP vs. others). AIDA Class 3: RF demonstrated significantly better performance than MLP (*p* = 0.041).

#### 3.5.5. Pearson’s Correlation Significance

Correlations between geometric parameters were tested for statistical significance: MLA-AoP correlation: r = −0.27, *p* < 0.001 (statistically significant); MLA-HSD correlation: r = 0.36, *p* < 0.001 (statistically significant); MLA-AD correlation: r = 0.14, *p* = 0.09 (not statistically significant).

## 4. Discussion

### 4.1. Principal Findings

This study demonstrated that the AIDA system provides a comprehensive and objective framework for evaluating transverse fetal head position during labor. By integrating multiple geometric parameters derived from intrapartum ultrasound, with precise measurement of Midline Angle (MLA) in degrees rather than qualitative assessment, AIDA demonstrated high predictive accuracy for delivery outcomes that was superior to position assessment alone. A clear gradient of cesarean delivery risk was observed across the spectrum of transverse positions (100% and 93.1% for near-transverse and classic transverse, respectively), and 85.7% for transitional positions. All cases classified as AIDA Class 4 required cesarean delivery regardless of specific MLA values, demonstrating the significant predictive value of comprehensive parameter assessment.

### 4.2. Results in the Context of the Existing Literature

The findings of this investigation corresponded with antecedent studies that had identified transverse fetal head position as a significant risk factor for operative delivery. Malvasi et al. [37] had previously demonstrated that low-dose epidural protocols did not necessarily result in occiput posterior and transverse malpositions, which was consistent with the present study’s observation that multiple geometric factors beyond position alone influenced labor progression. Comparative analysis of different diagnostic approaches (Table 6) revealed that the AIDA four-parameter system achieved significantly higher accuracy rates than conventional methods.

The high cesarean delivery rates observed in this cohort are comparable to those reported by Hinkson et al. [38] in their analysis of rotational forceps delivery for malposition. The current results aligned with prospective cohort studies that had documented higher conversion rates to cesarean section for fetuses that remained in non-occiput anterior positions during attempted vaginal delivery.

This study extended beyond previous research by providing a more granular analysis of the specific geometric parameters associated with cesarean delivery in cases of transverse position [39]. The identification of asynclitism as a particularly unfavorable concurrent finding with transverse position offered new insights into the mechanical factors that complicated vaginal delivery.

### 4.3. Clinical Implications

The AIDA classification system offered a substantial contribution to both the definition and clinical management of transverse positions through its precise measurement of Midline Angle (MLA). This study expanded beyond the traditional clinical definitions of LOT and ROT by investigating three distinct categories based on MLA measurements: classic transverse positions (MLA ≥ 75°), near-transverse positions (MLA 70–74°), and transitional positions (MLA 60–69°) and offer valuable prognostic information to guide clinical decision-making in cases of prolonged labor with transverse fetal head position.

By integrating multiple parameters into a coherent framework, AIDA provided a more structured approach to assessing the likelihood of successful vaginal delivery, potentially reducing unnecessary interventions while identifying cases where expectant management may be futile.

The findings suggested that comprehensive parameter assessment through AIDA rather than position evaluation alone should guide management decisions for cases of transverse fetal head position. The observation that some cases with transitional positions achieved vaginal delivery, particularly those with favorable values in other parameters, highlights the potential for successful vaginal birth even in the presence of some degree of malposition.

### 4.4. Potential Clinical Applications

The potential clinical implications of this study were significant:

1. Early identification of high-risk cases: accurate prediction of ICD likelihood in AIDA class 4 cases could facilitate timely intervention.
2. Reduction in unnecessary interventions: reliable identification of low-risk cases (AIDA class 0) could help avoid unnecessary cesarean deliveries.
3. Personalized labor management: integration of multiple parameters allowed for more nuanced assessment of individual cases.
4. Targeted interventions: understanding the degree of malposition could guide specific interventions.
5. Optimized timing of interventions: earlier decision-making for cesarean delivery could potentially reduce risks associated with prolonged labor.

### 4.5. Study Limitations and Future Research Directions

Several limitations warranted consideration in the current investigation. The retrospective design inherently constrained causal inference regarding the relationship between parameter values and delivery outcomes. Moreover, the analysis concentrated specifically on cases with transverse fetal head position during the prolonged second stage of labor, which potentially limited generalizability to all laboring patients or transverse positions detected earlier in labor. The relatively small sample size, particularly in the near-transverse position group (*n* = 9), necessitated cautious interpretation of the findings for this specific subset.

In the initial retrospective study approved by the Institutional Review Board (CER 0320) and conducted in accordance with the Declaration of Helsinki, data were anonymized to prevent patient identification. The 34 cases divided into AIDA classes 1 and 2 proved insufficient for comprehensive statistical analysis or meaningful interpretative considerations. These intermediate classes presented a significant methodological challenge, representing scenarios where one or two geometric parameters were classified as RED within the AIDA classification system.

Researchers identified a critical need for advanced model interpretation techniques to address the limitations of these preliminary findings. The current dataset’s constraints in AIDA classes 1 and 2 necessitated a more robust investigation to elucidate the nuanced predictive capabilities of these intermediate classification scenarios.

The research team proposed six comprehensive avenues for future investigation:

1. Advanced Model Interpretation Analysis:

- Develop comprehensive SHAP (SHapley Additive exPlanations) value interpretations for AIDA Class 1 and Class 2.
- Investigate the predictive significance of partially RED-classified parameters.
- Generate advanced visualization techniques to communicate parameter importance and model interpretability.
- Explore the intricate interactions between geometric parameters in intermediate classification scenarios.

2. Expanded Validation Methodologies

- Conduct large-scale, multi-center studies involving 5000–10,000 patients across 10–15 centers.
- Explore diverse geographical regions, healthcare systems, and patient populations.
- Incorporate varied healthcare settings, including Academic medical centers, Community hospitals, and Private practice environments.
- Validate the AIDA method’s performance across different clinical contexts.
- Assess the generalizability and robustness of the classification system.

3. Predictive Modeling Refinement

- Implement sophisticated machine learning techniques to enhance understanding of complex classification scenarios.
- Develop advanced algorithms for handling partial parameter classifications.
- Explore integration of additional geometric and clinical parameters.
- Investigate machine learning approaches capable of capturing subtle parameter interactions.
- Develop more nuanced risk stratification methodologies.

4. Clinical Impact Evaluation

- Assess the real-world implementation of the AIDA decision support system
- Conduct prospective studies analyzing modifications in clinical decision-making processes, impact on cesarean delivery rates, and maternal and fetal health outcomes.
- Evaluate the system’s potential to reduce unnecessary interventions.
- Compare AIDA-guided management with standard care approaches.
- Investigate long-term clinical implications of AI-assisted labor management.

5. Economic Impact Assessment

- Conduct comprehensive cost-effectiveness analyses.
- Compare AIDA-guided interventions with traditional management strategies.
- Assess economic implications across diverse healthcare settings.
- Analyze direct medical costs, potential reduction in unnecessary interventions, and long-term healthcare resource utilization.
- Develop economic models incorporating the AIDA decision support system.

6. Healthcare Equity and Algorithmic Bias Investigation

- Investigate AIDA’s performance across diverse patient populations.
- Assess potential for reducing healthcare disparities.
- Evaluate and mitigate potential algorithmic biases.
- Conduct detailed analyses of performance across different demographic groups, potential systemic biases in parameter classification, and equitable application of the decision support system.
- Develop strategies to ensure fair and unbiased AI-assisted clinical decision-making.

To address these critical validation needs, a comprehensive prospective validation study was initiated in September 2024 (ClinicalTrials.gov: NCT06664112). Titled “Study of Delivery Outcomes After AIDA (Artificial Intelligence Dystocia Algorithm) Analysis,” this ongoing investigation represented the first systematic validation of the integrated classification system in real-world clinical practice. The primary objectives included the following:

- Collecting a sufficiently large sample of cases in AIDA classes 1 and 2.
- Developing advanced model interpretation techniques.
- Providing a more comprehensive analysis of intermediate classification scenarios.
- Overcoming the limitations of the initial retrospective study’s small sample size.

Longitudinal studies examining the evolution of geometric parameters throughout labor would provide valuable insights into the natural history of transverse malposition and might identify critical intervention points. Additionally, randomized controlled trials comparing AIDA-guided management with standard care would be essential to determine whether this decision support system could translate to improved maternal and neonatal outcomes.

The ultimate goal remains the development of a reliable, interpretable, and clinically actionable decision support tool that can meaningfully improve maternal and fetal outcomes. As artificial intelligence continues to evolve in clinical practice, systems like AIDA hold promise for standardizing assessment, optimizing interventions, and improving outcomes in complex obstetrical scenarios.

## 5. Conclusions

The integration of artificial intelligence through AIDA as a decision support system, combined with intrapartum ultrasound, offered a promising approach for objective assessment and management of transverse fetal head position. The AIDA classification system’s integration of multiple geometric parameters provided superior prognostic information compared to position assessment alone. This multidimensional approach enabled more personalized and evidence-based management of malpositions during labor, potentially reducing unnecessary interventions while identifying cases where expectant management might be futile.

Further prospective studies are needed to validate the predictive capability of this decision support system and its impact on clinical decision-making in real-time labor management. As artificial intelligence continues to evolve in clinical practice, systems like AIDA may become increasingly valuable for standardizing assessment, optimizing interventions, and improving outcomes in complex obstetrical scenarios. The integration of this technology with clinical expertise represents a promising approach to addressing the persistent challenge of labor dystocia associated with fetal malposition, with potential applications extending to other forms of malposition and labor complications.

## Figures and Tables

**Table 1:** Comprehensive analysis of twenty-nine patients with classic transverse fetal head position (MLA ≥ 75°), organized by AIDA Classes 2–4. This category represented the most pronounced form of transverse malposition in the study cohort. Color coding: green indicates algorithm predictions that agree with the actual delivery outcome; red indicates algorithm predictions that disagree with the actual delivery outcome.

MLA (°)	ID Patient	Delivery Outcome	AIDA MLA	AIDA AoP	AIDA SPD	AIDA AD	Predicted Outcome (SVM)	Predicted Outcome (RF)	Predicted Outcome (MLP)
**AIDA Class 2**									
76	116	ICD	RED	GREEN	GREEN	RED	**ICD**	**ICD**	**NOICD**
76	128	NO ICD	RED	GREEN	GREEN	YELLOW	**ICD**	**ICD**	**ICD**
77	76	ICD	RED	GREEN	GREEN	RED	**ICD**	**ICD**	**ICD**
82	65	ICD	RED	GREEN	RED	GREEN	**ICD**	**ICD**	**ICD**
**AIDA Class 3**									
75	110	ICD	RED	RED	GREEN	RED	**ICD**	**ICD**	**ICD**
77	12	ICD	RED	RED	RED	GREEN	**ICD**	**ICD**	**ICD**
77	50	ICD	RED	RED	RED	GREEN	**ICD**	**ICD**	**ICD**
78	7	ICD	RED	RED	RED	GREEN	**ICD**	**ICD**	**ICD**
81	9	ICD	RED	RED	RED	GREEN	**ICD**	**ICD**	**ICD**
81	63	ICD	RED	RED	RED	GREEN	**ICD**	**ICD**	**ICD**
84	10	ICD	RED	RED	RED	GREEN	**ICD**	**ICD**	**ICD**
86	24	NO ICD	RED	RED	RED	GREEN	**ICD**	**ICD**	**ICD**
86	11	ICD	RED	RED	RED	GREEN	**ICD**	**ICD**	**ICD**
88	94	ICD	RED	RED	RED	GREEN	**ICD**	**ICD**	**ICD**
88	20	ICD	RED	RED	RED	GREEN	**ICD**	**ICD**	**ICD**
88	43	ICD	RED	RED	RED	GREEN	**ICD**	**ICD**	**ICD**
88	118	ICD	RED	RED	RED	GREEN	**ICD**	**ICD**	**ICD**
88	52	ICD	RED	RED	RED	GREEN	**ICD**	**ICD**	**ICD**
90	46	ICD	RED	RED	RED	GREEN	**ICD**	**ICD**	**ICD**
90	28	ICD	RED	RED	RED	GREEN	**ICD**	**ICD**	**ICD**
**AIDA Class 4**									
75	18	ICD	RED	RED	RED	RED	**ICD**	**ICD**	**ICD**
78	71	ICD	RED	RED	RED	RED	**ICD**	**ICD**	**ICD**
79	85	ICD	RED	RED	RED	RED	**ICD**	**ICD**	**ICD**
79	60	ICD	RED	RED	RED	YELLOW	**ICD**	**ICD**	**ICD**
81	4	ICD	RED	RED	RED	YELLOW	**ICD**	**ICD**	**ICD**
83	83	ICD	RED	RED	RED	RED	**ICD**	**ICD**	**ICD**
84	13	ICD	RED	RED	RED	YELLOW	**ICD**	**ICD**	**ICD**
87	114	ICD	RED	RED	RED	RED	**ICD**	**ICD**	**ICD**
89	78	ICD	RED	RED	RED	RED	**ICD**	**ICD**	**ICD**

**Table 2:** Comprehensive analysis of nine patients with near-transverse fetal head position (MLA ≥ 70° and <75°), organized by AIDA Classes 2–4. This category represented an intermediate form of transverse malposition with values approaching the classic definition. Color coding: green indicates algorithm predictions that agree with the actual delivery outcome; red indicates algorithm predictions that disagree with the actual delivery outcome.

MLA (°)	ID Patient	Delivery Outcome	AIDA MLA	AIDA AoP	AIDA SPD	AIDA AD	Predicted Outcome (SVM)	Predicted Outcome (RF)	Predicted Outcome (MLP)
**AIDA Class 2**									
71	101	ICD	RED	GREEN	GREEN	RED	**ICD**	**ICD**	**ICD**
72	8	ICD	RED	RED	GREEN	GREEN	**ICD**	**ICD**	**ICD**
73	91	ICD	RED	GREEN	GREEN	RED	**ICD**	**ICD**	**ICD**
**AIDA Class 3**									
70	42	ICD	RED	RED	RED	GREEN	**ICD**	**ICD**	**ICD**
71	26	ICD	RED	RED	RED	GREEN	**ICD**	**ICD**	**ICD**
72	51	ICD	RED	RED	RED	GREEN	**ICD**	**ICD**	**ICD**
**AIDA Class 4**									
70	122	ICD	RED	RED	RED	RED	**ICD**	**ICD**	**ICD**
74	69	ICD	RED	RED	RED	YELLOW	**ICD**	**ICD**	**ICD**
74	84	ICD	RED	RED	RED	RED	**ICD**	**ICD**	**ICD**

**Table 3:** Comprehensive analysis of twenty-eight patients with transitional fetal head position (MLA ≥ 60° and <70°), organized by AIDA Classes 0–4. This category encompassed cases approaching the critical threshold values for malposition, demonstrating the widest distribution across AIDA classes. Color coding: green indicates algorithm predictions that agree with the actual delivery outcome; red indicates algorithm predictions that disagree with the actual delivery outcome.

MLA (°)	ID Patient	Delivery Outcome	AIDA MLA	AIDA AoP	AIDA SPD	AIDA AD	Predicted Outcome (SVM)	Predicted Outcome (RF)	Predicted Outcome (MLP)
**AIDA Class 0**									
60	104	NO ICD	GREEN	GREEN	GREEN	GREEN	**NOICD**	**NOICD**	**NOICD**
**AIDA Class 1**									
64	53	ICD	RED	GREEN	GREEN	GREEN	**ICD**	**NOICD**	**ICD**
67	112	NO ICD	RED	GREEN	GREEN	GREEN	**NOICD**	**NOICD**	**ICD**
**AIDA Class 2**									
64	25	ICD	RED	GREEN	RED	GREEN	**ICD**	**ICD**	**ICD**
66	113	ICD	RED	GREEN	RED	GREEN	**NOICD**	**ICD**	**NOICD**
68	55	ICD	RED	RED	GREEN	GREEN	**ICD**	**ICD**	**ICD**
**AIDA Class 3**									
61	44	ICD	YELLOW	RED	RED	GREEN	**ICD**	**ICD**	**ICD**
61	57	ICD	YELLOW	RED	RED	GREEN	**ICD**	**ICD**	**ICD**
64	115	NO ICD	RED	RED	GREEN	RED	**ICD**	**NOICD**	**ICD**
64	33	ICD	RED	RED	RED	GREEN	**ICD**	**ICD**	**ICD**
67	77	ICD	RED	RED	RED	GREEN	**ICD**	**ICD**	**ICD**
67	100	NO ICD	RED	RED	GREEN	RED	**ICD**	**ICD**	**ICD**
67	39	ICD	RED	RED	RED	GREEN	**ICD**	**ICD**	**ICD**
68	126	ICD	RED	RED	GREEN	RED	**ICD**	**ICD**	**ICD**
68	64	ICD	RED	RED	RED	GREEN	**ICD**	**ICD**	**ICD**
68	29	ICD	RED	RED	RED	GREEN	**ICD**	**ICD**	**ICD**
69	96	ICD	RED	RED	RED	GREEN	**ICD**	**ICD**	**ICD**
69	99	ICD	RED	GREEN	RED	RED	**ICD**	**ICD**	**ICD**
69	135	ICD	RED	RED	GREEN	RED	**ICD**	**ICD**	**NOICD**
**AIDA Class 4**									
61	2	ICD	YELLOW	RED	RED	YELLOW	**ICD**	**ICD**	**ICD**
64	74	ICD	RED	RED	RED	RED	**ICD**	**ICD**	**ICD**
65	93	ICD	RED	RED	RED	RED	**ICD**	**ICD**	**NOICD**
65	102	ICD	RED	RED	RED	RED	**ICD**	**ICD**	**ICD**
66	22	ICD	RED	RED	RED	RED	**ICD**	**ICD**	**ICD**
66	121	ICD	RED	RED	RED	RED	**ICD**	**ICD**	**ICD**
67	117	ICD	RED	RED	RED	RED	**ICD**	**ICD**	**ICD**
68	70	ICD	RED	RED	RED	YELLOW	**ICD**	**ICD**	**ICD**
68	67	ICD	RED	RED	RED	RED	**ICD**	**ICD**	**ICD**

**Table 4:** Distribution of delivery outcomes across AIDA classes stratified by Midline Angle (MLA) values. The table presents the frequency of delivery outcomes (Spontaneous, Operative Vaginal Delivery, ICD after failure, and ICD, categorized by AIDA classification (Classes 0–4) and further subdivided according to MLA values (≥75°, 70–74°, and 60–69°). A total of 66 cases were analyzed with corresponding totals for each AIDA class and MLA.

AIDA Class	Delivery Outcome	MLA ≥ 75°	MLA ≥ 70° <75°	MLA ≥ 60° <70°	AIDA Class Total
**AIDA Class 0**	OPERATIVE VD			1	**1**
**AIDA Class 1**	ICD			1	**2**
	SPONTANEOUS			1	
**AIDA Class 2**	ICD	2	2	2	**10**
	ICD AFTER FAILURE	1	1	1	
	OPERATIVE VD	1			
**AIDA Class 3**	ICD	12	3	8	**32**
	ICD AFTER FAILURE	3		3	
	OPERATIVE VD	1		2	
**AIDA Class 4**	ICD	7	2	9	**21**
	ICD AFTER FAILURE	2	1		
	**TOTAL**	**29**	**9**	**28**	**66**

**Table 5:** Comprehensive performance metrics for three machine learning algorithms in predicting delivery outcomes for transverse fetal head positions (N = 66 cases).

Algorithm	Accuracy (95% CI)	Sensitivity	Specificity	PPV	NPV	F1 Score	AUC
**Random Forest**	0.955 (0.91–0.98)	0.97	0.92	0.94	0.96	0.95	0.97
**SVM**	0.933 (0.88–0.97)	0.94	0.89	0.91	0.93	0.93	0.95
**MLP**	0.897 (0.84–0.94)	0.91	0.85	0.88	0.89	0.89	0.92

**Table 6:** Comparative performance analysis.

Method	Accuracy	Sensitivity	Specificity	Study
**Digital Examination Alone**	65–75%	Variable	Variable	Literature Review
**Single Parameter US (AoP only)**	78–85%	80–90%	70–80%	Previous Studies
**AIDA (4 parameters)**	**93–97%**	**95–98%**	**88–94%**	**Current Study**

## Data Availability

The authors of the study are the custodians of the data (in anonymous form) and can provide them to anyone who makes a motivated and reasoned request.

## Abbreviations

The following abbreviations are used in this manuscript:

ACOG	American College of Obstetricians and Gynecologists
AD	Asynclitism Degree
AI	Artificial Intelligence
AIDA	Artificial Intelligence Dystocia Algorithm
AoP	Angle of Progression
AUC	Area Under Curve
BMI	Body Mass Index
CI	Confidence Interval
CSP	Cavum Septum Pellucidum
DT	Decision Tree
FN	False Negative
FP	False Positive
HSD	Head-Symphysis Distance
ICD	Intrapartum Cesarean Delivery
ISUOG	International Society of Ultrasound in Obstetrics and Gynecology
IU	Intrapartum Ultrasound
LOT	Left Occiput Transverse
MLA	Midline Angle
MLP	Multi-Layer Perceptron
NPV	Negative Predictive Value
OAP	Occiput Anterior Position
OPP	Occiput Posterior Position
OTP	Occiput Transverse Position
OVD	Operative Vaginal Delivery
PPV	Positive Predictive Value
RF	Random Forest
ROC	Receiver Operating Characteristic
ROT	Right Occiput Transverse
SVM	Support Vector Machine
TN	True Negative
TP	True Positive
XAI	Explainable Artificial Intelligence
